# Genomic Characterization of *Listeria monocytogenes* Isolated From Ready-to-Eat Meat and Meat Processing Environments in Poland

**DOI:** 10.3389/fmicb.2020.01412

**Published:** 2020-06-25

**Authors:** Monika Kurpas, Jacek Osek, Alexandra Moura, Alexandre Leclercq, Marc Lecuit, Kinga Wieczorek

**Affiliations:** ^1^Department of Hygiene of Food of Animal Origin, National Veterinary Research Institute, Pulawy, Poland; ^2^Institut Pasteur, Biology of Infection Unit, Paris, France; ^3^Institut Pasteur, National Reference Centre and WHO Collaborating Centre Listeria, Paris, France; ^4^Inserm U1117, Paris, France; ^5^Université de Paris, Necker-Enfants Malades University Hospital, Division of Infectious Diseases and Tropical Medicine, Institut Imagine, Paris, France

**Keywords:** *Listeria monocytogenes*, food, meat, cgMLST, WGS

## Abstract

*Listeria monocytogenes* is one of the major foodborne pathogens. Isolates of PCR-serogroups IIb (*n* = 17) and IVb (*n* = 31) recovered from food (*n* = 33) and food processing environment (*n* = 15) in Poland were characterized using whole genome sequencing. Most isolates belonged to Multi-Locus Sequence Type (MLST) ST2 (31.3%) and ST5 (22.9%). Core genome MLST (cgMLST) analysis classified isolates into seven sublineages (SL) and 25 different cgMLST types (CT). Consistent with the MLST results, most sublineages were SL2 and SL5. Eleven isolates harbored *aacA4* encoding resistance to aminoglycosides, three isolates harbored *emrC* (*n* = 3) and one *brcABC* (*n* = 1) encoding tolerance to benzalkonium chloride. Isolates belonging to SL5 CT2323 carried a so far unreported *inlB* allele with a deletion of 141 nucleotides encoding the β-repeat sheet and partially the GW1 domain of InlB. Comparison with publicly available genome sequences from *L. monocytogenes* isolated from human listeriosis cases in Poland from 2004 to 2013 revealed five common CTs, suggesting a possible epidemiological link with these strains. The present study contributes to characterize the diversity of *L. monocytogenes* in ready-to-eat (RTE) meat and meat processing environments in Poland and unravels previously unnoticed links with clinical cases in Europe.

## Introduction

*Listeria monocytogenes* is one of the most common foodborne zoonotic pathogens and the cause of listeriosis in human (Buchanan et al., 2017). The disease mostly occurs in elderly people, immunosuppressed patients, and pregnant women and their fetus/newborns (Swaminathan and Gerner-Smidt, 2007; Charlier et al., 2017). In immunosuppressed and older individuals, *L. monocytogenes* can cause septicaemia and meningitis, and in pregnant women induce miscarriage and neonatal listeriosis (Ramaswamy et al., 2007). *L. monocytogenes* may cause either sporadic cases or outbreaks and many of them have been linked to the consumption of contaminated food of animal origin (Buchanan et al., 2017; Glebícová et al., 2018). Different food products may be contaminated with *L. monocytogenes*, either at the production stage or during processing (Maury et al., 2019). According to a recent EFSA zoonotic report, depending on the ready-to-eat food category, from 0 to 3.18% samples were contaminated with *L. monocytogenes* in 2018 (European Food Safety Authority [EFSA], 2019). In the same year, 2,459 listeriosis cases were reported in the European Union, with a hospitalisation rate of 97.0% and a high fatality of 15.6% (European Food Safety Authority [EFSA], 2019). However, these values may not reflect the actual situation because European countries have different surveillance systems and the exhaustiveness of case notification and reporting also varies.

Molecular typing of *L. monocytogenes* is important for detecting clusters of human listeriosis cases as well as identifying the source of food contamination. Rapid, standardized and cheap methods are needed for screening many bacterial isolates as a first line for subsequent detailed characterization. PCR-serogrouping enables classification of *L. monocytogenes* into molecular serogroups which cover particular PCR-serogroups: IIa (including serovars 1/2a, 3a), IIb (1/2b, 3b, 7), IIc (1/2c, 3c), IVb (4b, 4d, 4e), and L (including serovars 4a, 4ab, 4c and other species of *Listeria sensu stricto*), respectively (Doumith et al., 2004). Most human infections are due to isolates of PCR-serogroups IVb, IIa, and IIb, which are responsible for over 95% of listeriosis cases, with *L. monocytogenes* of PCR-serogroup IVb causing more than half of these cases (Bergholz et al., 2018). Another molecular typing method is the multilocus sequence typing (MLST), which is based on the sequence variants of 7 housekeeping genes (Ragon et al., 2008). Recent advances in high-throughput sequence sequencing have enabled analysis of bacterial isolates at the whole genome level (Luth et al., 2018). The core genome MLST (cgMLST) typing method takes into account the sequence variation of 1,748 *L. monocytogenes* core genes, improving isolate discrimination and allowing a standardized comparison with isolate databases for outbreak investigations and surveillance (Moura et al., 2016, 2017).

Besides its increased discrimination power as compared to previous methods such as PFGE, whole genome sequencing enables simultaneous identification of antimicrobial resistance and virulence genes as well as other genetic determinants playing a role in *L. monocytogenes* infection (Moura et al., 2016).

Virulence of *L. monocytogenes* is associated with its ability to invade, multiply, and survive within host cells (Portnoy et al., 2002). Clones of *L. monocytogenes* differ in their pathogenic potential (Maury et al., 2016, 2019). Some *L. monocytogenes* are hypervirulent for humans are more often isolated from infection cases, whereas other isolates are hypovirulent, in part due to loss-of-function mutations in virulence genes (Glebícová et al., 2015; Maury et al., 2016, 2019). One of the most important virulence markers identified in *L. monocytogenes* is *Listeria* Pathogenicity Island-1 (LIPI-1), composed of six virulence genes regulated by PrfA, a transcriptional activator for more than 140 genes. These include *inlA* and *inlB* genes. InlA and InlB interaction with their respective receptor E-cadherin and c-Met, expressed by human epithelial cells, mediate *L. monocytogenes* internalisation into non-phagocytic cells (Lingnau et al., 1995). Premature stop codons (PMSC) in the *inlA* gene attenuate virulence in *L. monocytogenes* (Jacquet et al., 2004; Maury et al., 2016). Other proteins belonging to the internalin family are also engaged in *L. monocytogenes* virulence activity (Bierne et al., 2007).

It has been also shown that some *L. monocytogenes* clones survive better in the environment than others. This is the result of biofilm development and expression of tolerance to sanitizers, oxidative stress, alkaline or acid conditions and fridge temperature (Colegiogri et al., 2017). *L. monocytogenes* contains genomic islands Survival Stress Islet 1 (SSI-1) and Survival Stress Islet 2 (SSI-2) which are responsible for survival of the bacteria in suboptimal conditions commonly present in food processing environments (Hein et al., 2011). Tolerance to sanitizers and disinfectants such as benzalkonium chloride, is often encoded by *emrC* and *bcrABC* genes (Dutta et al., 2013), present on mobile genetic elements that may be easily transmitted between different *L. monocytogenes* (Dutta et al., 2013; Kremer et al., 2017; Maury et al., 2019).

In Poland, limited information is available regarding *L. monocytogenes* diversity in food and food processing environment. The objectives of the present study were: (i) the molecular characterisation of *L. monocytogenes* of PCR-serogroups IIb and IVb isolated from ready-to-eat meat and meat processing environment in Poland using WGS and (ii) the comparison of the obtained sequences with genomes of *L. monocytogenes* isolated from human listeriosis cases.

## Materials and Methods

### Bacterial Strains

A total of 48 *L. monocytogenes* isolates classified by PCR-serogrouping (as detailed below) into PCR-serogroup IIb (*n* = 17) and PCR-serogroup IVb (*n* = 31) were selected for the whole genome sequencing and genomic analyses. They were isolated in official laboratories between 2014 and 2017 from different kinds of ready-to-eat (RTE) food of animal origin (e.g., ham, sausages or meat) (*n* = 33) and from food processing environment (*n* = 15), using the standard ISO 11290-1:1996+A1:2004 (ISO, 1996). The isolates were originated from 9 voivodeships (administrative regions) of Poland and sent to the laboratory of the Department of Hygiene of Food of Animal Origin, National Veterinary Research Institute in Pulawy. Subsequently, the isolates were cultured on TSYEA (tryptone soya yeast extract agar; Oxoid, United Kingdom) at 37 ± 1°C for 18–4 h and the isolates were identified biochemically at species level using API *Listeria* (Biomerieux, France) according to the manufacturer’s instructions. The confirmed *L. monocytogenes* were stored at −70°C for further analysis.

### Determination of *L. monocytogenes* PCR-Serogroups

*L. monocytogenes* were cultured on TSYEA at 37 ± 1°C for 18–24 h and a loopful of bacteria was transferred into 100 μl of TRIS buffer (A&A Biotechnology, Poland). DNA was extracted using the Genomic Mini protocol (A&A Biotechnology) with the modification by adding 15 μl of lysozyme (10 mg/ml; Sigma-Aldrich, United States) for 30 min at 37°C. *L. monocytogenes* isolates were molecularly typed for PCR-serogroups using multiplex PCR as previously described (Doumith et al., 2004). All isolates used in the study are listed in Supplementary Table S1.

### DNA Isolation, Library Preparation and Sequencing

DNA extraction was performed according to the modified Genomic Mini protocol as described above with additional modification by suspension of DNA in 100 μl of DNAse, RNAse free water (MP Biomedicals, United States) at 75°C. DNA quality and concentration were measured by NanoDrop and Qubit three (Thermo Fisher Scientific, United States). DNA library was prepared by the Nextera XT DNA Library Preparation Kit (Illumina, United States) according to the manufacturer’s instruction. DNA was sequenced on either Illumina Mi-seq (*n* = 40) or NextSeq500 (*n* = 8) sequencing platforms, respectively. All sequences trimmed with fqCleaner v.3.0 (Alexis Criscuolo, Institut Pasteur) and assembled with SPAdes v.3.11 with the automatic kmer selection (Bankevich et al., 2012). Assembly quality was assessed using the number of contigs, N50 and L50 metrics.

### MLST and cgMLST Characterization

MLST (7 loci; Ragon et al., 2008) and cgMLST profiles (1,748 loci; Moura et al., 2016) were extracted from the assemblies using the BLASTN algorithm (Altschul et al., 1990) as previously described (Moura et al., 2016). MLST profiles were classified into sequence types (ST) and grouped into clonal complexes (CCs) as previously described (Ragon et al., 2008). cgMLST profiles were grouped into cgMLST types (CTs) and sublineages (SLs), using the cut-offs of 7 and 150 allelic mismatches, respectively, as previously described (Moura et al., 2016). The cgMLST profiles obtained in this study were also compared with those from 55 PCR-serogroup IVb and 8 PCR-serogroup IIb publicly available genome sequences of *L. monocytogenes* isolated from patients in Poland (Kuch et al., 2018). All calculations were made with the method described above. Allele numbers, STs, CCs, CTs, and SLs were determined according to the *Listeria* sequence typing database and its tools available on BIGSdb-*Lm* platform^1^ (Jolley and Maiden, 2010; Moura et al., 2016). MLST and cgMLST profile comparisons were done using Bionumerics 7.6 software (Applied Maths, Belgium) with single linkage algorithm ignoring missing values in pairwise comparisons. Dendrograms were visualized using iTOL^2^ (Letunic and Bork, 2016).

### Virulence, Antimicrobial Resistance and Stress-Related Genes

Identification of antimicrobial resistance, virulence and benzalkonium chloride tolerance genes (listed in Supplementary Table S1) was done using BIGSdb-*Lm* platform. Single gene alignments were performed using BLAST^3^ and MEGA7 softwares (Kumar et al., 2015).

## Results

### MLST Analysis

**FIGURE 1:**
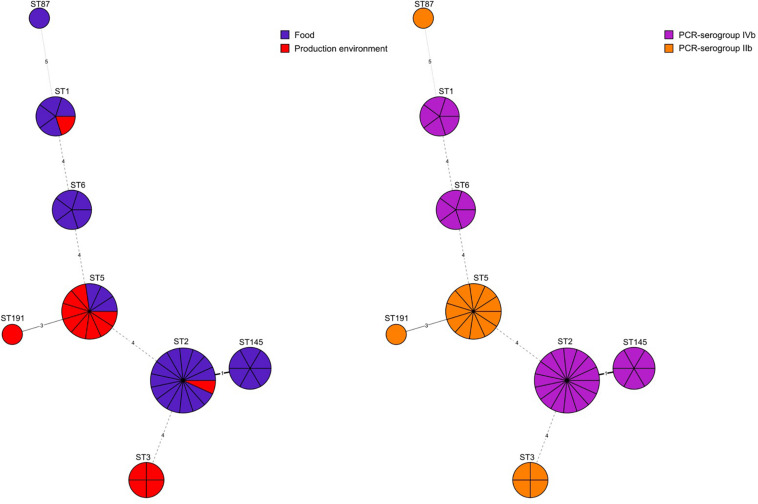
Minimum spanning tree analysis based on MLST allelic profiles of 48 *L. monocytogenes* isolates. Each circle represents a sequence type (ST). The numbers on the connecting lines illustrate the numbers of target genes with different alleles. The sources of isolates and their serogroups are distinguished by the colors.

Eight different MLST sequence types were identified: ST2 (31.2%, *n* = 15 isolates), ST5 (23.0%, *n* = 11), ST145 (12.5%, *n* = 6), ST1 (10.4%, *n* = 5), ST6 (10.4%, *n* = 5), ST3 (8.3%, *n* = 4), ST187 (2.1%, *n* = 1), and ST191 (2.1%, *n* = 1). *L. monocytogenes* belonging to PCR-serogroup IIb contained isolates classified to four sequence types (ST3, ST5, ST87, and ST191) whereas isolates of PCR-serogroup IVb were classified to 4 other STs (ST1, ST2, ST6, and ST145) (Figure 1 and Supplementary Table S1). Moreover, *L. monocytogenes* were grouped into seven clonal complexes (CC1, CC2, CC3, CC5, CC6, CC87, and CC191). Isolates from food processing environment mostly belonged to ST5/CC5 (8 out of 15 isolates) whereas the isolates of food origin were mainly classified to ST2/CC2 (14 out of 33 isolates) (Supplementary Table S1).

### cgMLST Analysis

**FIGURE 2:**
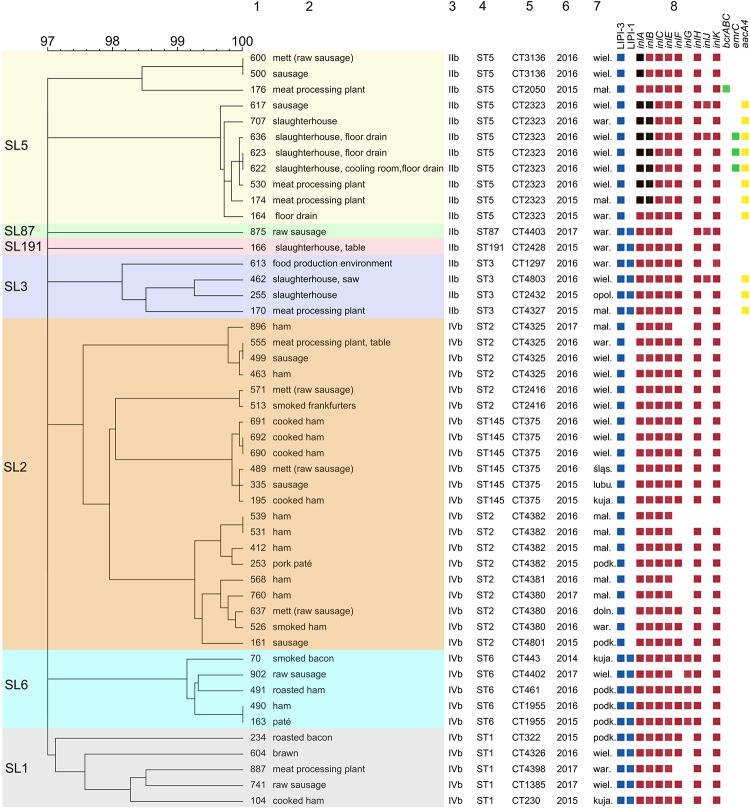
Dendrogram representing clustering of *L. monocytogenes* PCR-serogroups IIb and IVb by cgMLST using single linkage clustering method. Degeneracy cut off value were 97% of similarity. Column: 1 – strain ID, 2 – source, 3 – PCR-serogroup, 4 – ST type, 5 – cgMLST type, 6 – year of isolation, 7 – place of isolation, 8 – detected genes. Color squares show presence of *Listeria* Pathogenicity Islands 1 and 2 (blue), internalins (orange), benzalkonium chloride resistance (green) and aminoglycosides resistance genes (yellow), respectively. Black squares indicate the presence of genes with truncation. Full metadata information are available in Supplementary Table S1. F, food, FPE, food production environment.

Based on the cgMLST analysis, *L. monocytogenes* isolates were classified into 25 different CTs and grouped into seven SLs: SL2 (43.7%, *n* = 21 isolates), SL5 (22.9%, *n* = 11), SL1 (10.4%, *n* = 5), SL6 (10.4%, *n* = 5), SL3 (8.3%, *n* = 4), SL87 (2.1%, *n* = 1), SL191 (2.1%, *n* = 1) (Figure 2 and Supplementary Table S1).

Among isolates originating only from food (*n* = 19), 6 different cgMLST types comprising more than one isolate were identified. The most prevalent one, cgMLST type L1-SL2-ST145-CT375, covered 6 isolates obtained from 4 different regions (kujawsko-pomorskie, lubuskie, śla̧skie, wielkopolskie voivodeships). The remaining cgMLST types were L1-SL2-ST2-CT4382 (4 isolates), L1-SL2-ST2-CT4380 (3 isolates), L1-SL5-ST5-CT3136 (2 isolates), L1-SL2-ST2-CT2416 (2 isolates), and L1-SL6-ST6-CT1955 (2 isolates) (Figure 2 and Supplementary Table S1).

Isolates exclusively from food processing environment (*n* = 7) were classified into seven different cgMLST types (Supplementary Table S1), mostly to SL5 and SL3.

Two CTs comprised isolates from both food and food processing environment. The first cgMLST type, L1-SL5-ST5-CT2323 (*n* = 8), consisted of one isolate from meat and 7 environmental isolates, including 4 isolates from floor drains in meat production areas. They originated from 3 voivodeships (Polish administrative regions) and were collected during two years (2015–2016). The second cgMLST type, L1-SL2-ST2-CT4325 (*n* = 4), consisted of 3 isolates from food and one isolate from food processing environment, isolated in 3 voivodeships during two-year period (2016, 2017) (Supplementary Table S1).

### Comparison of Isolates From Food and Food Production Environment With Human *L. onocytogenes*

**FIGURE 3:**
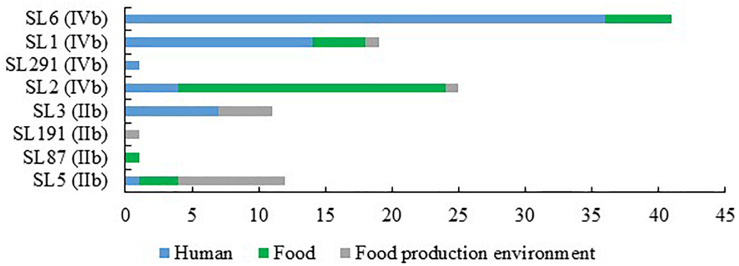
Number of *L. monocytogenes* sublineages among isolates of PCR-serogroups IIb and IVb tested in the study.

**FIGURE 4:**
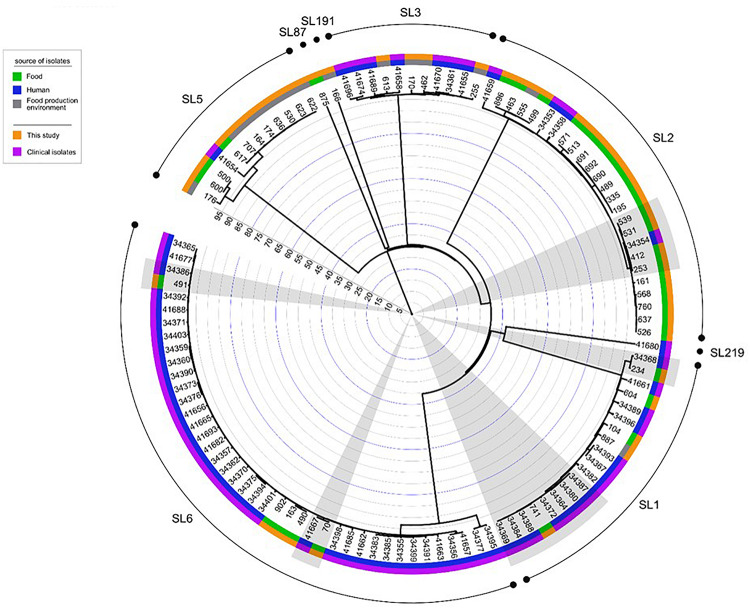
Dendrogram representing the comparison of *L. monocytogenes* isolates analyzed in this study with the published genomes of strains of PCR-serogroups IIb and IVb recovered in Poland from humans with listeriosis. Analysis was made on the base of cgMLST profiles using a single linkage clustering method. Gray shadows highlight the five groups of isolates with the 99.6% similarity, isolated from both humans and food. Clinical isolates were described by Kuch et al. (2018). Rings info, from the outer side: sublineages, source of isolates, number of isolates, the scale. Full metadata information is available in Supplementary Table S1.

**FIGURE 5:**
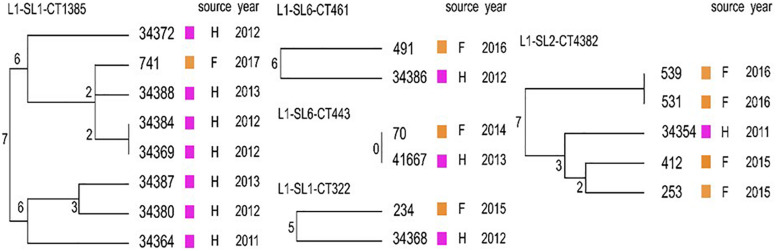
Detailed view of the five cgMLST types identified in this study common to Polish clinical cases described by Kuch et al. (2018). Dendrograms were built based on cgMLST profiles using a single linkage clustering method. In the nodes, the number of allelic distances is indicated. F – food; H – humans.

When comparing the present isolates with publicly available ones obtained from human listeriosis cases in Poland collected from 2004 to 2013, it was noticed that *L. monocytogenes* responsible for infections mostly belonged to sublineages SL3, SL1 and SL6, while the majority of the isolates of food and food production environment origins were classified into SL2 and SL5 sublineages (Figure 3). Moreover, five cgMLST types, belonging to sublineages SL1, SL2, and SL6, were found among *L. monocytogenes* isolated from both humans and food (Figure 4). In detail, isolate 741 recovered in 2017 from food (cgMLST type L1-SL1-ST1-CT1385) displayed 2–7 allelic difference to a cluster of seven isolates of human origin isolated during 2011–2013. Within cgMLST type L1-SL2-ST2-CT4382, 4 isolates (ID539, ID531, ID412, and ID253) collected from food between 2015 and 2016 also showed 2–7 allelic difference to the clinical isolate (ID34354) obtained in 2011. Moreover, among *L. monocytogenes* strains of L1-SL6-ST6-CT461 and L1-SL1-ST1-CT322 cgMLST types, isolated from food between 2015 and 2016 as well as from clinical cases in 2012, five and six allelic mismatches were also identified. Additionally, one food isolate (ID70, cgMLST L1-SL6-CT443 type from 2014) did not reveal any allelic differences with the isolate of clinical origin (ID41667) from 2013 (Figure 5).

### Virulence Factor Genes

Identification of selected virulence genes showed that the pathogenicity islands LIPI-1 and LIPI-3 were present in 48 (100%) and 15 (31.3%) isolates tested, respectively. LIPI-3 was identified in 10 isolates of PCR-serogroup IVb (5 SL1 and 5 SL6) and 5 isolates classified to PCR-serogroup IIb (4 SL3 and 1 SL191). None of the isolates was positive for LIPI-4 genes. The internalin gene family members *inlA*, *inlB*, *inlC*, *inlE*, *inlF*, and *inlJ* were found in all isolates tested, whereas the *inlH* and *inlK* genes were present in 47 (97.9%) and 46 (95.8%) isolates, respectively. *inlG* was found only in 5 isolates of PCR-serogroup IVb classified to SL6 (Supplementary Table S1).

**FIGURE 6:**
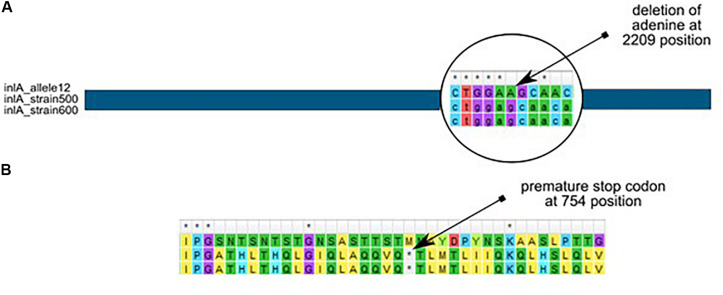
Adenine deletion in the newly identified *inlA* PMSC type 31 mutation in *L. monocytogenes* L1-SL5-ST5-CT3136 (PCR-serogroup IIb), as compared to *inlA* allele 12 (from SL5) retrieved from BIGSdb-*Lm*. **(A)** – nucleotide alignment, **(B)** – amino acid alignment.

Further analysis revealed that almost all *L. monocytogenes* SL5 isolates (10 out of 11; 90.9%, belonging to two different cgMLST types), harbored a premature stop codon (PMSC) mutation in *inlA* (Figure 3). In L1-SL5-ST5-CT2323 (8 isolates), the *inlA* PMSC type 1 (T1818A; Nightingale et al., 2005) was detected, whereas in L1-SL5-ST5-CT3136 (2 isolates) a new PMSC was identified (new allele *inlA*_302, termed PMSC type 31). This new mutation was caused by the deletion of adenosine in position 2209, resulting in a shorter peptide of 753 amino acids lacking InlA anchoring motif to peptidoglycan (Figure 6).

In addition to an *inlA* PMSC, isolates belong to L1-SL5-ST5-CT2323 cgMLST type also carried a new *inlB* allele (allele 170), with a deletion of 141 nucleotides encoding the β-repeat sheet and partially the GW1 domain of the InlB protein.

### Benzalkonium Chloride Tolerance and Other Tolerance Genes

Genes conferring benzalkonium chloride tolerance (*bcrABC* or *emrC*) were detected in 4 (8.3%) out of 48 analyzed *L. monocytogenes* (Supplementary Table S1). The *bcrABC* gene cassette was noticed in one isolate (L1-SL5-ST5-CT2050) originating from a meat processing plant. The *emrC* gene was harbored by 3 isolates (all L1-SL5-ST5-CT2323) obtained from the floor drains in slaughterhouses.

The SSI-1 gene cluster encoding the stress factor was detected among isolates belonging to L1-SL3-ST3 (4 isolates from CT1297, CT4803, CT2432, CT4327), L1-SL5-ST5 (3 isolates from CT2050, CT3136), and L1-SL191-ST191-CT2428 (1 isolate) cgMLST types, respectively. None of the examined isolates harbored the SSI-2 stress marker (Supplementary Table S1). Additionally, the isolates had the genes responsible for resistance to lysozyme (*pdgA*) and bile salts (*bsh*), which belong to core genes present in all *L. monocytogenes* (Supplementary Table S1).

### Antimicrobial Resistance Genes

WGS identification of antimicrobial resistance genes revealed that in all 48 *L. monocytogenes* isolates tested, four intrinsic resistance genes were present, as expected for core genes: *fosX* (resistance to fosfomycin), *lmo0919* (lincosamides), *norB* (quinolones), and *sul* (sulfonamides). The acquired *aacA4* gene (encoding resistance to aminoglycosides) was found in some serogroup IIb isolates, belonging to 4 different cgMLST types: L1-SL5-ST5-CT2323 (8 isolates), L1-SL3-ST3-CT2432, L1-SL3-ST3-CT4327, and L1-SL3-ST3-CT4803 (1 isolate each), mainly obtained from food production environment in 4 voivodeships (Figure 2).

## Discussion

A total of 48 *L. monocytogenes* isolates originating from ready-to-eat meat and meat processing environment were typed and characterized for the presence of virulence, antimicrobial resistance and stress response genetic determinants. MLST typing revealed that *L. monocytogenes* isolates were classified to 6 clonal complexes. Among them, there were CC1, CC2, and CC6 (total of 31, 64.6% isolates), previously described as human infection-associated hypervirulent clones in France (Maury et al., 2016). Several other studies have also shown that *L. monocytogenes* of clonal complexes CC1, CC2, CC4, and CC6 are often isolated from patients with listeriosis (Bergholz et al., 2018; Kuch et al., 2018). In the present investigation most of the isolates classified to these hypervirulent clonal complexes were recovered from RTE food. This confirms the previous findings that meat products may be a potential source of *L. monocytogenes* associated with human infections (Buchanan et al., 2017). Furthermore, the present analysis also showed that isolates belonging to five cgMLST types found in food (LI-SL1-ST1-CT1385, LI-SL1-ST1-CT322, LI-SL2-ST2-CT4382, LI-SL6-ST6-CT461, LI-SL6-ST6-CT443) were previously identified among human listeriosis isolates in Poland (Kuch et al., 2018) and other European countries (Denmark and France) (Moura et al., 2016, 2017). The close genetic distance between isolates within each cgMLST suggests a possible epidemiological link.

Most of *L. monocytogenes* isolates of PCR-serogroup IIb (11 of 17, 64.7%) belonged to clonal complex CC5 and were mainly of food processing environment origin (8 isolates). Moreover, 4 of these strains belonged to L1-SL5-ST5-CT2323 cgMLST type, were collected from floor drains at different environmental conditions present in cooling rooms and slaughterhouses. It was previously shown that floor drains may be “hotspots” on the spreading pathways of *L. monocytogenes* in food processing plants (Dzieciol et al., 2016). Several studies demonstrated that isolates belonging to CC5 and sublineage SL5 survive better in food processing environment than others, probably due to the presence of pLM80 plasmid with the *bcrABC* gene cassette responsible for tolerance to benzalkonium chloride, a very common compound of sanitizers using in food industry (Muhterem-Uyar et al., 2018). In the present study, the *bcrABC* marker was identified only in one isolate classified to LI-SL5-ST5-CT2050 cgMLST type of PCR-serogroup IIb. Another gene connected with benzalkonium chloride resistance (*emrC*) was found in 3 *L. monocytogenes* isolates, all belonging to LI-SL5-ST5-CT2323. These results suggest that some *L. monocytogenes* strains tested in the present study harbor resistance to adverse environmental conditions and may survive in food processing plants.

The results of the present study also showed that 11 *L. monocytogenes* strains from PCR-serogroup IIb, assigned to cgMLST types L1-SL5-ST5-CT2323, L1-SL3-ST3-CT4327, and L1-SL3-ST3-CT2432, harbored the *aac4A* gene which encodes resistance to aminoglycosides. It has been previously shown that this gene was present in different bacterial species and can be transferred between bacteria, including *L. monocytogenes* identified in food and food processing environment (Van et al., 2007; Sacha et al., 2012).

In the current study, the differences in the presence of virulence factors *inlA* and *inlB* among *L. monocytogenes* isolates belonging to PCR-serogroups IIb and IVb were found. The premature stop codon in the *inlA* gene was detected in 10 isolates classified to PCR-serogroup IIb and sequence type ST5 whereas this gene marker was not identified in any of the strains of PCR-serogroup IVb. The distribution of PMSC dependent on *L. monocytogenes* PCR-serogroups were also described by other authors (Jennison et al., 2017; Su et al., 2019). It has been previously shown that *L. monocytogenes* with this mutation are less virulent for humans, because truncation of this gene leads to the lack of the anchoring motif which is necessary for the cross-linking of InlA to peptidoglycan (Lebrun et al., 1996; Jonquieres et al., 1998). As described before, strains with cgMLST type L1-SL5-ST5-CT2323 have type 1 mutation in the *inlA* gene which was also confirmed in the present study (Glebícová et al., 2015). Moreover, a novel type of mutation identified in this gene was found in two isolates belonging to ST5 but classified to L1-SL5-ST5-CT3136.

The virulence gene *inlB* was identified in all 48 *L. monocytogenes* tested in the present study, including the isolates of cgMLST L1-SL5-ST5-CT2323 type with the deletion of 141 nucleotides. However, the impact of this mutation in InlB function has not been tested, but it may alter InlB association to *L. monocytogenes* cell surface. Strains with the same deletion, isolated from mushroom production environment, have been identified previously (Muruesan et al., 2015). It has been described that different isoforms of the *inlB* gene have an impact on signaling pathways and invasiveness of *L. monocytogenes* (Wałecka-Zacharska et al., 2015; Sobyanin et al., 2017; Chalenko et al., 2019). Of note, differences in the sequences of the *inlB* gene among strains of wild animals and humans were observed, which may suggest that mutations in this gene may have an impact on the invasiveness of *L. monocytogenes* (Zaytseva et al., 2007). Moreover, all our *L. monocytogenes* with deletion in the *inlB* gene also had PMSC mutation within the *inlA* marker which may suggest that these isolates are hypovirulent. Furthermore, among 8 isolates classified to ST3, ST5, and ST191 sequence types, the SSI-1 marker responsible for the adaptation of isolates to the extreme environmental conditions (resistance to bile salts and acids) was detected (Hein et al., 2011).

## Conclusion

Whole genome sequencing of *L. monocytogenes* of PCR-serogroups IIb and IVb isolated from food and food processing environment allowed the characterization of the isolates and the ability to look at genetic traits associated with virulence and resistance to antimicrobials and environmental conditions. MLST and cgMLST analyses allowed determination of the CCs, SLs and CTs for molecular characterization and comparison of the present isolates with strains originated from different sources and countries. It was shown that some *L. monocytogenes* from food and from human listeriosis cases in Poland were classified to 5 common cgMLST types. Our study confirms food of animal origin as a source of pathogenic *L. monocytogenes* for humans.

## Data Availability Statement

The datasets generated for this study can be found in the NCBI database under BioProject PRJNA629756 and in BIGSdb-*Lm*.

## Author Contributions

MK, JO, and KW contributed to the conception and design of the study. KW and JO planned the study. MK and AM performed the experiments and performed the bioinformatic analyses. MK, JO, AM, AL, ML, and KW analyzed the data and drafted the manuscript. All authors critically read and approved the final version of the manuscript.

## Conflict of Interest

The authors declare that the research was conducted in the absence of any commercial or financial relationships that could be construed as a potential conflict of interest.

## Footnotes

1 https://bigsdb.pasteur.fr/listeria

2 https://itol.embl.de

3 https://blast.ncbi.nlm.nih.gov/Blast.cgi
